# Targeting IGF1R signaling for brain aging and Alzheimer’s disease

**DOI:** 10.31491/apt.2022.12.103

**Published:** 2022-12-29

**Authors:** Joo Young Park, Martin Darvas, Warren Ladiges

**Affiliations:** a Department of Comparative Medicine, School of Medicine, University of Washington, Seattle, WA, USA.; b Department of Laboratory Medicine and Pathology, School of Medicine, University of Washington, Seattle, WA 98195, USA.

**Keywords:** IGF1R signaling, IGF1R^R407H^ variant, brain aging, cognition, Alzheimer’s disease

## Abstract

The role of IGF1R signaling in the brain and its relationship to aging and neurological dysfunction is controversial. Because it was shown that low IGF1R activity consistently improved myocardial bioenergetics and function in hearts from aging mice, but not hearts from young mice, it was of interest to investigate this relationship in brain aging. We used CRISPR technology to develop a mouse model with targeted replacement of mouse IGF1R with the equivalent of the human R407H (IGF1R^R407H^) variant enriched in centenarians with a reduction in IGF1R protein activity. Middle-aged mice show improved cognitive performance thus possibly modeling IGF1R signaling in the aging brain, similar to what was reported in the aging heart. Because Alzheimer’s disease (AD) is an age-related disease, specific IGF1R^R407H^ pathways could be therapeutic targets in mice with AAV vector-based AD as well as for overall brain aging.

Signaling of insulin/insulin-like growth factor-1 (IGF1) through the IGF1-receptor (IGF1R) is central to processes that modulate aging and lifespan in model organisms [1–2], as well as mortality and morbidity in humans [3]. IGF1R signaling controls cellular growth and metabolism through a wide variety of downstream pathways. A recent study by Abdellatif *et al*. [4] showed that low IGF1R activity consistently improved myocardial bioenergetics and function in hearts from aging mice, but not hearts from young mice, in an autophagy-dependent manner. They concluded that the relationship between IGF1R signaling and cardiac health is not linear, but biphasic, since mice with low IGF1R signaling exhibited poor cardiac function early in life, while young mice with increased IGF1R signaling showed a superior cardiac function that progressively declined with increasing age. Moreover, analyses of serum IGF1 levels and various clinical diseases lend further support for such antagonistic pleiotropy [5]: protection of young individuals with high IGF1 levels and increased disease risk in older individuals with higher IGF1 levels. While much work on IGF1R has focused on peripheral organs, there is reason to assume the same mechanism occurs in the brain. It has been shown that increased IGF1R signaling was apparent in postmortem brains of Alzheimer’s disease (AD) patients [6], suggesting that a long-term increase in activity is associated with the progression of AD neuropathology. Genetic ablation of IGF1R in neurons of aging mice can protect against neuroinflammation and memory impairment induced by A beta oligomers [7]. However, the role of IGF1R signaling in the brain and its relationship to aging and neurological dysfunction is controversial. For example, Tazearslan *et al*. [8] reported that age-related astrocyte dysfunction caused by diminished IGF1R signaling may contribute to the pathogenesis of AD and other age-related cognitive impairments. A possible explanation for the opposite effects to be in play may be that different downstream pathways respond differently with increasing age. Evidence to support this possibility showed that Ashkenazi Jewish centenarians were found to be enriched with variants in the *IGF1R* gene [9]. Individuals carrying two specific variants, A37T and R407H, had a significant reduction in IGF1R protein and its phosphorylation levels.

Based on these observations, we used CRISPR technology to develop a mouse model with a targeted replacement of mouse IGF1R with the equivalent of the human R407H (IGF1R^R407H^) variant [10]. The objective was to establish a precision animal model that could help provide definitive answers to the role of IGF1R signaling in aging, including age-associated conditions such as cardiac aging and neurodegeneration. Since IGF1RR407H disrupts phosphorylation levels of ERK and AKT but not IGF1R-IGF1 binding, these specific pathways (and probably others) can be interrogated using this newly developed mouse line. Thus, we can study how a single variant may down-regulate IGF1R signaling in a more precise pathway-specific manner to provide a protective environment to delay brain aging and prevent the development of neurological conditions such as Alzheimer’s disease (AD). Is this mouse line then a model of the biphasic signaling by IGF1R in the aging brain similar to what Abdellatif *et al*. [4] reported in the aging heart? Perhaps, but more work is needed. We have not observed any adverse phenotypic effects at younger ages but do see an improved cognitive performance at older ages compared to wild-type littermates.

These published and unpublished observations provide the rationale to develop therapeutic targets for the IGF1RR407H variant to prevent or delay brain aging. There is support for IGF1R as a target for therapeutic intervention of aging. Huffman *et al*. [11] showed that 18-month-old female but not male mice treated with a monoclonal antibody (mAb) targeting IGF1R (L2-Cmu, Amgen Inc) had improved health span and increased median lifespan by 9 percent along with a reduction in neoplasms and inflammation compared to controls. These effects were achieved at advanced ages, suggesting that IGF1R mAbs could represent a promising therapeutic candidate to delay aging. In a second study, picropodophyllin (PDP), a selective, competitive, and reversible inhibitor of IGF1R that is brain penetrant, was given to a mouse model of AD amyloid neuropathology (APP/PS1 Tg mice) daily for 7 days IP at 1 mg/kg/day [12]. PDP attenuated levels of Aβ40 and 42 and decreased microgliosis and p-tyrosine in the hippocampus.

Since AD is an age-related disease, drugs that prevent or slow down brain aging should also be effective in helping resist the development of AD neuropathology and the associated severe cognitive impairment. So far, there has been no single therapeutic compound that has been shown in clinical trials to have significant beneficial reversible effects even though preclinical animal studies were promising. There may be a disconnect between currently available mutant FAD mouse models and the ability to predict clinical outcomes. In most of the transgenic lines, a significant increase in APP production begins early in life possibly in utero, which may trigger consequences that alter aging and the rate of aging, and likely does not mimic the biochemical changes observed in AD. A further complication is the lack of the mixed amyloid and Tau pathology that characterizes AD. We have developed an aging mouse model of AD neuropathology using an AAV-based transfer of Aβ42 and mutant MAPT (tau P301L) [13], which allows us to test drugs in a more realistic preclinical manner. Middle-aged and older aged mice can be obtained from the United States National Institute on Aging Aged Rodent Colony and used in drug testing, without having to wait several years for young mice to age.

Drugs can be tested in older mice over several months so different therapeutic strategies can readily be carried out. However, a high throughput drug screening system for IGF1R^R407H^ or other IGF1R variants has yet to be developed so candidate drugs are somewhat limited. RNA sequencing could help determine whether to target specific downstream pathways. Regardless, there is a rationale for identifying drugs that target IGF1R signaling based on the beneficial aspects of significant reductions in activity at older ages. A preclinical mouse model is in place to speed up the process of moving from preclinical testing to clinical trials for treating and preventing AD (Figure 1).

## Figures and Tables

**Figure 1.**
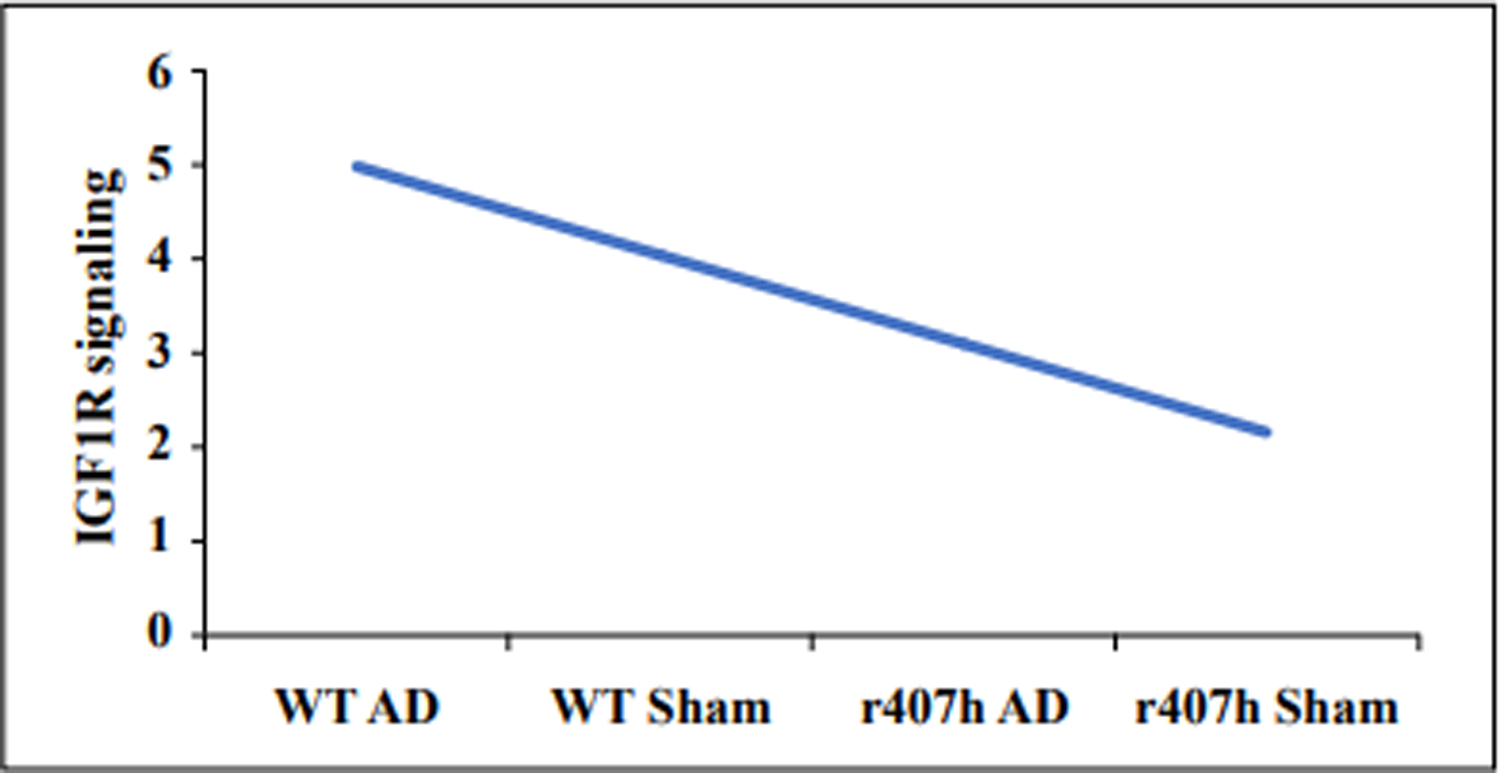
A hypothetical example of the effect of the IGF1R^R407H^ variant on IGF1R signaling on a scale of 1 to 5 (low to high) in 22-month-old C57BL/6 mice with or without AAV vector-transferred Alzheimer’s disease. Abbreviations: AD = AAV Aβ42+tauP301L; r407h = the IGF1R variant; Sham = AAV vector only; WT = wild-type littermates.
